# Perceived stress and resilience in family caregivers of patients with mental illness : relationship and correlates

**DOI:** 10.1192/j.eurpsy.2023.2307

**Published:** 2023-07-19

**Authors:** S. Kolsi, N. Charfi, I. Gassara, R. Feki, S. Omri, N. Smaoui, L. Zouari, J. Benthabet, M. Maalej, M. Maalej

**Affiliations:** psychiatry c department, Hedi Chaker University Hospital Center, Sfax, Tunisia

## Abstract

**Introduction:**

Family members play an important role in the life of many adults with mental disorders and are under considerable amounts of stress that may affect caregiver’s physical health, quality of life and resilience.

**Objectives:**

The present study aimed to explore the relationship between the perceived stress and the resilience levels among caregivers of patients with mental illness and to identify their associated factors.

**Methods:**

This was a cross-sectional, descriptive and analytical study conducted on caregivers of patients suffering from mental illness. It was conducted in the outpatient psychiatry department at the university hospital of Sfax (Tunisia), during september 2021.

We used the Connor–Davidson Resilience Scale to assess resilience and the Perceived Stress Scale (PSS-10) to assess the level of stress. High scores indicate high resilience and perceived

**Results:**

The sample included 34 family caregivers of patients with mental illness. The mean age was 47.47 years (SD=12.4 years) and the sex ratio (M/F) was 1.42.

The mean resilience score of caregivers was 42.85 and the mean perceived stress score was 24.94 (SD=6.36).

The score of resilience correlated negatively with the score of perceived stress among family caregivers (r=-0.751 ; p=0.0001).

The Caregivers with low socioeconomic level were more likely to have a low resilience score (p=0.004) and to have high stress levels (p=0.04).

The level of perceived stress increased significantly in case of long duration of providing care (r=0.697 ; p= 0.001), the presence of stressful events (p=0.029) and the presence of agressive behaviors committed by patients (p= 0.001). However, the level of resilience decreased significantly in those same cases (p=0.001; p=0.002; p=0.0001 respectively)

**Conclusions:**

Our findings suggest that high level of perceived stress among familiy caregivers impact negatively their capacity of resilience. So, interventions targeting stress related to stressful events and violence committed by patients in their family environment should be integrated to increase the caregivers’resilience.

**Disclosure of Interest:**

None Declared

